# Supplementary material for: Mechanism for Reducing the Horizontal Transfer Risk of the Airborne Antibiotic-Resistant Genes of Escherichia coli Species through Microwave or UV Irradiation
Source: Int J Environ Res Public Health. 2022 Apr 4;19(7):4332. doi: 10.3390/ijerph19074332 (PMC8998220; doi:10.3390/ijerph19074332)
Supplement: Supplementary file 1 [file ijerph-19-04332-s001.zip › ijerph-1618444-supplementary.pdf]

Mechanism for Reducing the Horizontal Transfer Risk of the Airborne Antibiotic-Resistant Genes of *Escherichia coli* Species through Microwave or UV Irradiation

Azhar Ali Laghari [1], Liming Liu [1], Dildar Hussain Kalhoro [2], Hong Chen [1,*] and Can Wang [1,3,*]

1 School of Environmental Science and Engineering, Tianjin University, Tianjin 300350, China; azharlaghari18@hotmail.com (A.A.L.); liuliming_1998@163.com (L.L.)
2 Department of Veterinary Microbiology, Faculty of Animal Husbandry and Veterinary Sciences, Sindh Agriculture University, Tandojam 70050, Pakistan; drdildarkalhoro@gmail.com
3 Tianjin Key Laboratory of Indoor Air Environmental Quality Control, Tianjin 300350, China
* Correspondence: chenhong_0405@tju.edu.cn (H.C.); wangcan@tju.edu.cn (C.W.)

Text S1. The extraction of intracellular DNA (iDNA)

Figure S1. The effects of MW, and UV at various power

Table S1. Primers information.

Text S1. The extraction of intracellular DNA (iDNA)

10 mL of 0.12 M NaH2PO4 (pH = 8.0) and 1.0 g of PVPP were added to 2 mL of sample. The mixture was thoroughly shaken at 2500 rpm for 10 min at 25 °C with a vortex, and then centrifuged at 10,000 g for 10 min at 4 °C. To remove eDNA, the supernatant was vacuum filtered using a 0.22 m sterilized polytetrafluoroethylene (PTFE) filter membrane. The filter membrane was re-suspended in 10 mL of 0.12 M NaH2PO4 (pH = 8.0) and discarded, after which the supernatant was centrifuged at 10,000 g for 10 min at 4 °C and poured supernatantaway to remove any remaining eDNA. 4 mL DNA extraction buffer (100 mM Tris-HCl, 100 mM Na2-EDTA, 100 mM Na3PO4, 1% CTAB, 0.05 mgmL-1 proteinase K, and 1.5 M NaCl) and 0.5 g glass beads (Φ1 mm) were added to the above-mentioned precipitates. The re-suspension was shaken at 2500 rpm for 10 min before being uniformly mixed with 4 mL of 10% SDS. The mixture was frozen in nitrogen for 1 min and immediately placed in water baths at 60 °C for 20 min using the freeze-thawing method, which was repeated three times to break up the cell wall and release more iDNA, then centrifuged at 13,000 g for 20 min at 4 °C to obtain supernatant A and precipitates. The precipitates were resuspended in 4 mL of DNA extraction buffer and agitated at 2500 rpm for 10 min, after which they were evenly added to 4 mL of 10% SDS and placed in water baths at 37 °C for 120 min. To obtain the supernatant B, the mixture was put in water baths at 60 °C for 20 min and centrifuged at 13,000 g for 20 min at 4 °C.This process results in cell lysis, and protein and polysaccharide precipitation, while protecting iDNA from damage. The iDNA was disinfected from the supernatant A and B using the DNeasy Power Water Kit (Qiagen GmbH, Hilden, Germany) according to the manufacturer's instructions.

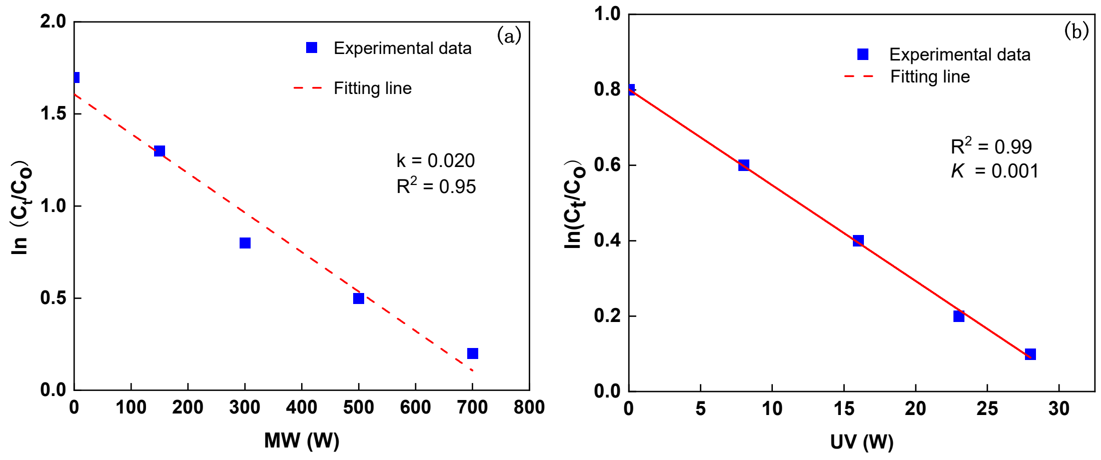

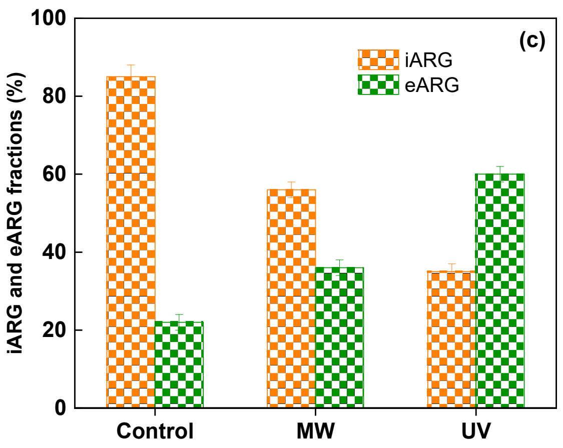

Figure S1. The effects of MW, and UV at various power (a) pseudo-first-order kinetic under MW, (b) pseudo-first-order kinetic under MW, (c) eARGs and iARGs fractions.

Table S1. Primers used in this study for qPCR analyses.

Gene name	Primer	Primer Sequence (5′→3′)	Amplicon Size (bp)	References
*Sul*1	FW	CGCACCGGAAACATCGCTGCAC	163	Xi et al., 2009
	RV	TGAAGTTCCGCCGCAAGGCTCG		
*Sul*2	FW	TCCGGTGGAGGCCGGTATCTGG	191	Pei et al., 2006
	RV	CGGGAATGCCATCTGCCTTGAG		
*Sul*3	FW	TCCGTTCAGCGAATTGGTGCAG	128	Pei et al., 2006
	RV	TTCGTTCACGCCTTACACCAGC		

[a] FW, forward; RV, reverse.

References

1. Pei, R.T.; Kim, S.C.; Carlson, K.H.; Pruden, A. Effect of River Landscape on the sediment concentrations of antibiotics and corresponding antibiotic resistance genes (ARG). *Water Res.* **2006**, *40*, 2427–2435. https://doi.org/10.1016/j.watres.2006.04.017.
2. Xi, C.W.; Zhang, Y.L.; Marrs, C.F.; Ye, W.; Simon, C.; Foxman, B.; Nriagu, J. Prevalence of antibiotic resistance in drinking water treatment and distribution systems. *Appl. Environ. Microbiol.* **2009**, 75, 5714-5718. https://doi.org/10.1128/AEM.00382-09.
